# Preoperative ANemiA among the elderly undergoing major abdominal surgery (PANAMA) study: Protocol for a single-center observational cohort study of preoperative anemia management and the impact on healthcare outcomes

**DOI:** 10.1097/MD.0000000000010838

**Published:** 2018-05-25

**Authors:** Hairil Rizal Abdullah, Yilin Eileen Sim, Yi Tian Mary Sim, Ecosse Lamoureux

**Affiliations:** a Department of Anaesthesiology, Singapore General Hospital; b DukeNUS Medical School, Singapore, Singapore; c Academic Medicine Research Institute (AMRI), DukeNUS Medical School, Singapore, Singapore.

**Keywords:** elderly, major abdominal surgery, preoperative anemia

## Abstract

**Introduction:**

Preoperative anemia and old age are independent risk factors for perioperative morbidity and mortality. However, despite the high prevalence of anemia in elderly surgical patients, there is limited understanding of the impact of anemia on postoperative complications and postdischarge quality of life in the elderly. This study aims to investigate how anemia impacts elderly patients undergoing major abdominal surgery in terms of perioperative morbidity, mortality and quality of life for 6 months postoperatively.

**Methods and analysis:**

We will conduct a prospective observational study over 12 months of 382 consecutive patients above 65 years old, who are undergoing elective major abdominal surgery in Singapore General Hospital (SGH), a tertiary public hospital. Baseline clinical assessment including full blood count and iron studies will be done within 1 month before surgery. Our primary outcome is presence of morbidity at fifth postoperative day (POD) as defined by the postoperative morbidity survey (POMS). Secondary outcomes will include 30-day trend of POMS complications, morbidity defined by Clavien Dindo Classification system (CDC) and Comprehensive Complication Index (CCI), 6-month mortality, blood transfusion requirements, days alive out of hospital (DaOH), length of index hospital stay, 6-month readmission rates and Health Related Quality of Life (HRQoL). HRQoL will be assessed using EuroQol five-dimensional instrument (EQ-5D) scores at preoperative consult and at 1, 3, and 6 months.

**Ethics and dissemination:**

The SingHealth Centralised Institutional Review Board (CIRB Ref: 2017/2640) approved this study and consent will be obtained from all participants. This study is funded by the National Medical Research Council, Singapore (HNIG16Dec003) and the findings will be published in peer-reviewed journals and presented at academic conferences. Deidentified data will be made available from Dryad Repository upon publication of the results.

## 1 Introduction

Preoperative anemia is associated with poor postoperative outcomes, such as increased mortality, length of stay, intensive care unit admissions, and complications including acute kidney injury, stroke and postoperative infection.^[1–14]^ It is also associated with perioperative blood transfusion,^[15,16]^ which is independently associated with increased risk of perioperative morbidity and mortality.^[6,17]^

Old age is another independent risk factor for perioperative morbidity, mortality and increased healthcare resource utilization,^[18–21]^ as the elderly may have limited physiologic reserve and higher prevalence of unrecognized cardiovascular disease that renders them more vulnerable to the stress of surgery compared to younger patients.

Put together, old age and anemia may have a compounding effect, which is concerning as the prevalence of anemia increases with age in both the general and surgical population. In a large cohort of surgical patients in a tertiary hospital in Singapore, 45.0% of elderly patients aged above 70 years had preoperative anemia, which is much higher than the overall incidence of 27.8% of anemia in the surgical population.^[22,23]^ Indeed, initial studies show that anemic elderly undergoing noncardiac surgery have increased risk of postoperative 30-day mortality and cardiac event.^[13]^ Studies in the nonsurgical population have also suggested that anemia may negatively affect physical function, cognitive performance, mood, and quality of life of the elderly person.^[24–27]^ However, the impact of preoperative anemia on elderly patients in terms of postsurgical recovery and short- and medium-term quality of life beyond the hospitalization period is not currently known.

In light of the above, we have designed an observational study to examine the impact of preoperative anemia on elderly surgical patients undergoing major abdominal surgery. The study will encompass a large spectrum of outcome measures that are collected during hospitalization and after discharge up to 6 months. The primary aim of this study is to determine the presence of morbidity at 5th postoperative day (POD) using the postoperative morbidity survey (POMS). Our secondary aim is to determine the trend of complications within 30 postoperative days (POD) as defined by POMS, 30-day morbidity as defined by Clavien Dindo Classification system (CDC) and Comprehensive Complication Index (CCI), perioperative transfusion requirements, length of hospital stay, 6-month mortality, 6-month readmission rates, Days alive and out of Hospital (DaOH) at 30 days and 6 months, and Health Related Quality of Life (HRQoL) outcomes as assessed by the EuroQol five-dimensional instrument (EQ-5D) questionnaire. Data from this study will encourage clinicians to investigate and manage perioperative anemia, and guide future intervention. Furthermore, the findings could inform perioperative shared decision-making process among the elderly surgical patients.^[28]^

## 2 Methods and analysis

### 2.1 Overview of study design

**Figure 1:**
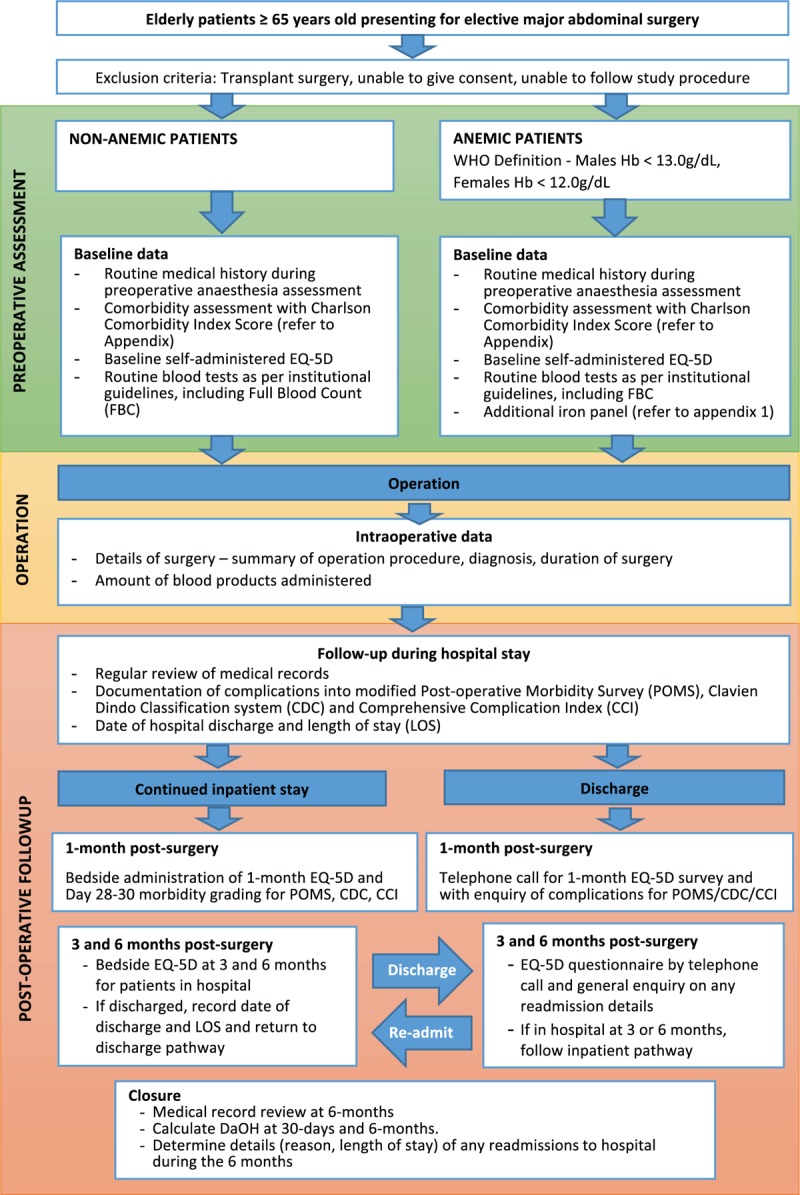
Study schedule. CCI = Comprehensive Complication Index, CDC = Clavien Dindo Classification system, DaOH = Days Alive and Out of Hospital, EQ-5D = EuroQol five-dimensional instrument, Hb = hemoglobin; LOS = length of stay, POMS = Postoperative Morbidity Survey, WHO = World Health Organization.

This is a single-centre prospective observational study on patients aged 65 years and above, who are undergoing elective major abdominal surgery in Singapore General Hospital (SGH), a tertiary public hospital, over a 12-month period. All patients will be followed for six months from the date of operation. We plan to recruit a minimum of 382 patients. Figure 1 shows the study schedule.

### 2.2 Patient recruitment

Recruitment will take place during the patient's preoperative evaluation clinic visit in SGH. This typically occurs within 4 weeks before the surgery. The study coordinators will explain in private and obtain written consent from willing participants. Inclusion criteria: aged 65 years or older and ability to sign informed consent; undergoing elective major abdominal surgery for benign or malignant disease (major defined as procedures expected to last more than 2 hours, or with an anticipated blood loss greater than 500 mL). Exclusion criteria: Organ transplant surgery (specifically, patients who are admitted to the hospital for an organ transplant; patients who had previous transplants but have been discharged can be included). Inability to comprehend and/or perform study procedures due to language barrier or other organic causes. Table 1 shows the eligibility criteria of the study.

**Table 1:**
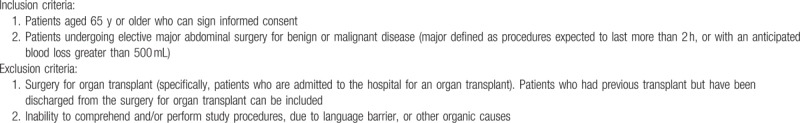
Inclusion and exclusion criteria.

### 2.3 Sample size

The sample size was calculated based on a pilot study of this protocol conducted in our institution. In our sample of 41 patients, the incidence of anemia was 51%. The presence of postoperative morbidities as defined as Post-Operative Morbidity Survey (POMS) score ≥1 on day 5 was 7/21 (33%) in nonanemic group and 10/20 (50%) in anemic group. Thus, to have a power of 90% to detect the difference of 17% (50–33%) significantly, the number of patients needed in each arm would be 159 patients, hence 318 patients. Taking into account a potential dropout rate of 20% at 6-month follow-up, our targeted sample size would be 382.

### 2.4 Baseline assessment

Patient's demographic data and presence of significant comorbidities will be recorded from the routine preoperative assessment clinical notes. Comorbidity burden will be recorded and analyzed using Charlson Comorbidity Index scores.^[29]^ The Charlson Comorbidity Index is a method of weighting comorbid conditions to measure burden of disease and case mix. The index has been widely used in both clinical and health research and has been validated for its ability to predict mortality in various disease subgroups, including colorectal cancer, general surgical and elderly patients.^[30–34]^

We will take preoperative Full Blood Count as per normal clinical pathway. The last laboratory value within 1 month before the index surgery will be used to determine preoperative anemia status. Anemia will be diagnosed based on the World Health Organisation gender-based stratification of anemia severity.^[35]^ Mild anemia will be defined as hemoglobin concentration of 11 to 12.9 g/dL in males and 11 to 11.9 g/dL in females. Moderate anemia for both genders will be hemoglobin concentration between 8 and 10.9 g/dL and severe anemia will be defined as <8.0 g/dL. As per our institution guidelines, patients with anemia will undergo further investigations such as iron studies (serum ferritin, iron, total iron binding capacity, and transferrin saturation).

Self-administered baseline measures of EQ-5D will be taken during the preoperative assessment visit. The research coordinator will guide patients in filling out the questionnaire. The EQ-5D is a generic measure of HRQoL comprising of a Visual Analogue Scale (VAS) for self-rating of general health (a score of 100 is the best possible general health status) and 5 domains evaluating mobility, self-care, usual activities, pain/discomfort, and anxiety/depression. The EQ-5D has been used widely in Singapore to examine HRQoL among different disease groups and population norms have been established for EQ-5D in Singapore.^[36]^ Questionnaires will be made available in English, Simplified Chinese, Malay and Tamil to suit the primary language of the patient.

### 2.5 Intraoperative data

Intraoperative data such as surgical details (summary of operation procedure, diagnosis, surgical duration) and amount of blood products administered will be obtained via hospital electronic records.

### 2.6 Study outcomes and follow-up

Our primary outcome is the presence of morbidity at the 5th postoperative day (POD) as indicated according to the presence of any POMS-defined complications. POMS was developed to classify in-hospital complications that a patient may encounter after surgery into nine domains—neurological, hematological, pain, gastrointestinal, wound, cardiovascular, pulmonary, infectious, and renal.^[37]^ We will then continue to monitor the trend of POMS-defined complications for the first 30 postoperative days as part of our secondary outcomes. Patients will have their POMS complications graded on POD 3, 5, 7–8, 14–15, 21–22, and 28–30, with flexible ranges for the last three periods of assessment to ease the logistics of follow up.

In addition to POMS, postoperative complications up to POD 30 will be graded using the CDC and CCI.^[37,38]^ CDC grades the severity of surgical complications based on the therapy used to treat the complication, and is scored based on the highest grade of complication.^[38]^ The CCI at Day 30 then takes into account all Clavien Dindo complications incurred and gives different weightage to each according to their severity.^[39,40]^

Other secondary health-care outcomes include perioperative blood transfusion requirement, defined as blood transfusion requirements from 1 month before surgery up to day of discharge from surgery, length of hospital stay for surgery, readmission to the hospital for any reason within 6 months of date of surgery, 6-month mortality, Days Alive and Out of Hospital (DaOH), and HRQoL outcomes.

DaOH was devised as a tool to evaluate endpoints in clinical trials in heart failure^[41–43]^ and has been further validated as a measure for postsurgical outcomes in Australia.^[44]^ It is a patient-centric outcome that reflects the burden of length of stay of index admission, readmissions, and early death after surgery. The DaOH index in this study will be calculated by summating the total amount of time spent by the patient in acute-care hospital for the surgery and in rehabilitation in step-down hospitals after the surgery, as well as any readmissions back to hospitals, within 30 days and 6 months after the surgery. As time spent at home and out of hospital is highly valued by patients, DaOH will be a good representative of the overall postoperative experience. For this study, we estimate the DaOH index at 30 days and 6 months to reflect the short and long-term effect of anemia. The information will be obtained during telephone follow ups with the patient when we collect information about their HRQoL. Further readmission details such as length of stay will be determined by subsequent medical record review.

HRQoL outcomes are assessed by determining EQ-5D scores at baseline, 1, 3, and 6 months postoperatively. Apart from the baseline EQ-5D score obtained at the preoperative assessment clinic, the EQ-5D score at 1, 3, and 6 months will be obtained by telephone interview of the patients still hospitalized at 30 days after surgery will have EQ-5D questionnaire administered at the bedside together with postoperative day 28 to 30 POMS. If they are hospitalized at 3 or 6 months postoperatively, the EQ-5D questionnaire will be administered by their bedside.

A list of the study outcomes is shown in Table 2.

**Table 2:**
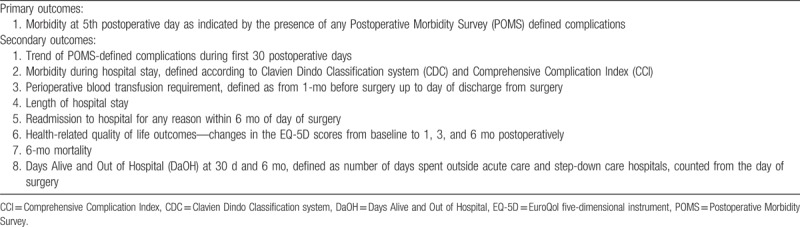
Primary and secondary outcomes.

### 2.7 Quality control

All data will be monitored and reviewed by the Principal Investigator (PI) or co-investigators (Co-I). The PI and Co-I will provide training to the research coordinators with regards to the administration and interpretation of various scoring systems used in this study. If there is any disagreement in the classification of existing comorbidities or postoperative complications, the PI or Co-I will be involved to reach an agreeable resolution.

The participants can withdraw their participation from the study at any time. While patients do not need justification for withdrawing from the study, the reasons for their withdrawal will be recorded. In addition, patients who are lost to follow up during the study will have their baseline characteristics such as demographic data, preoperative comorbidity including anemia, baseline EQ-5D and intraoperative details analyzed to see if there are significant differences between those who were lost to follow up and those who completed the study follow up.

As this is an observational study, and there is no interaction between the study team members and the patient's primary physicians that can potentially alter their management, the patients are not exposed to additional risks due to their participation in this project. Hence, no compensation is anticipated to be necessary.

### 2.8 Data management

Participant data confidentiality will be maintained throughout the study. Each patient will be assigned a unique study-related index number upon recruitment. This study-related index number will be used on the case report form. The key to the index numbers will be stored in a separate document and access restricted to the principal and co-investigator.

All research data will be entered into a hard-copy case report form and the deidentified data will be reentered in the institution's Research Electronic Data Capture (REDCap) System, which is a centralized secured data management platform. Records generated in this study for all participants, such as the patient's written informed consent, EQ-5D assessment forms and postoperative complication assessment forms, as well as the Singhealth Centralised Institutional Review Board (CIRB) records and other regulatory documentation will be retained by the PI and be accessible for inspection by authorities. Only study team members will have access to the research data.

Compliant with CIRB policy, both soft and hard copies of the research data will be kept for at least 6 years.

### 2.9 Statistical methods

#### 2.9.1 Statistical analyses

The demographic characteristic, baseline clinical features, and outcomes between anemic and nonanemic patients will be compared using the Chi-square test for categorical variables and the independent samples *T* test or Mann–Whitney *U* test for continuous variables. For binary outcomes of interest such as presence of any POMS-defined complication at 5th POD, presence of Clavien Dindo complications of grade 3 and above, death and readmission within 6 months, and any perioperative blood transfusion, univariate, and multivariate logistic regression analysis will be performed to determine the impact of relevant clinical variables as well as anemia. For continuous outcomes, such as length of stay, DaOH, CCI, and EQ-5D, we will perform linear regression with the General Linear Model or quantile regression where appropriate to estimate the slope coefficients of preoperative anemia and other relevant clinical variables. *P* values and 95% confidence intervals will be presented.

#### 2.9.2 Analysis of EQ-5D data

The EQ-5D has been used widely in Singapore to examine HRQoL among different disease groups and population norms have been established for EQ-5D in Singapore, allowing a cost-utility analysis in determining quality-adjusted life years.^[36,45–47]^ Changes of EQ-5D scores from the baseline value to values at 3 and 6 months will be compared between patients with and without preoperative anemia. Missing EQ-5D data for patients known to be alive and not hospitalized at the time point being analyzed will be imputed using the “last observation carried forward” method, for which at least one previous follow-up value is required. If no preceding values were available, the patient will be excluded from that analysis. For hospitalized patients unable to complete the questionnaires, or if the patient had died between the last time point and the planned time point, the worst reply will be used for the imputation, that is, 3 for the EQ-5D subquestions and 0 for the EQ-5D VAS score. Similar approach has been used in previous anemia-related study reporting HRQoL via EQ-5D.^[48]^

### 2.10 Data monitoring

Data monitoring will be performed by the Principal Investigator (PI) and co-investigators (Co-I). The practices are also subjected to audit and monitoring by the Division of Research at SGH as well as the CIRB. No safety monitoring will be performed as this is not an interventional trial. There are no plans for interim analyses due to the relatively low sample size and anticipated rapid recruitment rate. Data monitoring is independent from the sponsor and competing interests.

## 3 Ethics and dissemination

This study will be conducted in accordance with the Singapore Good Clinical Practice Guidelines, which is based on the principles enshrined in the Declaration of Helsinki. This study has been approved by the Singhealth Centralised Institutional Review Board (CIRB) (CIRB Ref: 2017/2640). Any protocol modification will be submitted to the CIRB for approval before implementation. Results of this study will be presented at international conferences and submitted to a peer-reviewed journal. Deidentified data will be made available from Dryad Repository upon publication of the results.

## Acknowledgments

We would like to thank our statistician Dr. Hao Ying of Singhealth Health Services Research Unit for reviewing the statistics of the protocol.

## Author contributions

**Conceptualization:** Hairil Rizal Abdullah, Yilin Eileen Sim, Yi Tian Mary Sim, Ecosse Lamoureux.

**Data curation:** Hairil Rizal Abdullah, Yilin Eileen Sim, Yi Tian Mary Sim, Ecosse Lamoureux.

**Formal analysis:** Hairil Rizal Abdullah, Yilin Eileen Sim, Yi Tian Mary Sim, Ecosse Lamoureux.

**Funding acquisition:** Hairil Rizal Abdullah, Yilin Eileen Sim, Yi Tian Mary Sim, Ecosse Lamoureux.

**Investigation:** Hairil Rizal Abdullah, Yilin Eileen Sim, Yi Tian Mary Sim, Ecosse Lamoureux.

**Methodology:** Hairil Rizal Abdullah, Yilin Eileen Sim, Yi Tian Mary Sim, Ecosse Lamoureux.

**Project administration:** Hairil Rizal Abdullah, Yilin Eileen Sim, Yi Tian Mary Sim, Ecosse Lamoureux.

**Resources:** Hairil Rizal Abdullah, Yilin Eileen Sim, Yi Tian Mary Sim, Ecosse Lamoureux.

**Software:** Hairil Rizal Abdullah, Yilin Eileen Sim, Yi Tian Mary Sim, Ecosse Lamoureux.

**Supervision:** Hairil Rizal Abdullah, Yilin Eileen Sim, Yi Tian Mary Sim, Ecosse Lamoureux.

**Validation:** Hairil Rizal Abdullah, Yilin Eileen Sim, Yi Tian Mary Sim, Ecosse Lamoureux.

**Visualization:** Hairil Rizal Abdullah, Yilin Eileen Sim, Yi Tian Mary Sim, Ecosse Lamoureux.

**Writing – original draft:** Hairil Rizal Abdullah, Yilin Eileen Sim, Yi Tian Mary Sim, Ecosse Lamoureux.

**Writing – review & editing:** Hairil Rizal Abdullah, Yilin Eileen Sim, Yi Tian Mary Sim, Ecosse Lamoureux.
